# The Prevalence of Anemia in Children Aged 6–23 Months and its Correlates Differ by District in Kapilvastu and Achham Districts in Nepal

**DOI:** 10.1016/j.cdnut.2023.100063

**Published:** 2023-03-03

**Authors:** Lindsey M. ocks, Naveen Paudyal, Sabrina Lundsgaard, Lila Bikram Thapa, Nira Joshi, LZuguo Mei, Ralph D. Whitehead, Maria Elena D. Jefferds

**Affiliations:** 1 Department of Health Sciences, College of Health and Rehabilitation Sciences: Sargent College, Boston University, Boston, MA, United States; 2 Department of Global Health, School of Public Health, Boston University, Boston, MA, United States; 3 Nutrition Section, United Nations Children’s Fund, Kathmandu, Nepal; 4 Nutrition Section, Family Welfare Division, Ministry of Health and Population, Kathmandu, Nepal; 5 New ERA, Kathmandu, Nepal; 6 Nutrition Branch, Division of Nutrition, Physical Activity, and Obesity, Centers of Disease Control and Prevention, Atlanta, GA, United States

**Keywords:** anemia, iron deficiency, inflammation, malnutrition, child health

## Abstract

**Background:**

Analyses of predictors of anemia or malnutrition often pool national or regional data, which may hide variability at subnational levels.

**Objectives:**

We sought to identify the risk factors for anemia in young Nepali children aged 6–23 mo in 2 districts: Kapilvastu and Achham.

**Methods:**

This is an analysis of two cross-sectional surveys that were conducted as part of a program evaluation of an infant and young child feeding and micronutrient powder intervention that included anemia as a primary outcome. Baseline and endline surveys in each district (in 2013 and 2016) included hemoglobin assessments in *n* = 4709 children who were representative of children 6–23 mo in each district. Log-binomial regression models accounting for the survey design were used to estimate univariable and multivariable prevalence ratios for risk factors at multiple levels—underlying, direct, and biological causes. Average attributable fractions (AFs) for the population were calculated for significant predictor biomarkers of anemia in multivariable models.

**Results:**

In Accham, the prevalence of anemia was 31.4%; significant predictors included child’s age, household asset ownership, length-for-age *z*-score, inflammation (CRP concentration > 0.5 mg/L; α-1 acid glycoprotein concentration > 1 mg/mL), and iron deficiency (serum ferritin concentration < 12 μg/L with BRINDA-inflammation adjustment). In Kapilvastu, the prevalence of anemia was 48.1%; significant predictors included child’s sex and ethnicity, wasting and weight-for-length z-score, any morbidity in the previous 2 wk, consumption of fortified foods, receipt of multiple micronutrient powder distributions, iron deficiency, zinc deficiency (nonfasting serum zinc concentration of <65 μg/dL in the morning and that of <57 μg/dL in the afternoon), and inflammation. In Achham, average AFs were 28.2% and 19.8% for iron deficiency and inflammation, respectively. Average AFs for anemia in Kapilvastu were 32.1%, 4.2%, and 4.9% for iron deficiency, zinc deficiency, and inflammation, respectively.

**Conclusions:**

The prevalence of anemia and its risk factors varied between districts, with inflammation contributing to a greater share of anemia in Achham than in Kapilvastu. The estimated AF for iron deficiency was around 30% in both districts; iron-delivering interventions and multisectoral approaches to anemia are warranted.

## Introduction

Globally, an estimated 40% of children aged 6–59 *mo* suffered from anemia in 2019 [1]. Anemia is characterized by a low hemoglobin concentration and/or RBC count that is insufficient to meet an individual’s physiological needs [2], and it is associated with an increased risk of morbidity and mortality in women and children [3,4], as well as impaired cognitive and behavioral development in children [5]. Anemia and iron deficiency are highly prevalent in Nepal, where one-third of the children aged 6–23 *mo* have anemia and 46.6% of the children aged 6–23 *mo* have iron deficiency [6].

Interventions to reduce anemia in vulnerable populations heavily focus on the consumption of iron and/or multiple micronutrients through supplementation or fortification (including industrial fortification, biofortification, and home-fortification approaches). It has been estimated that ∼50% of the worldwide burden of anemia is due to iron deficiency [7]; however, the etiology and pathophysiology of anemia are complex and likely vary across contexts [8]. Analyses of predictors of anemia often pool data on a national or regional level [9,10], which may hide important variability at the subnational level. In the current analysis, we used 2 large surveys that are representative of children aged 6–23 *mo* in 2 districts in Nepal to evaluate district-specific predictors of anemia.

## Methods

### Survey design, sampling, and data collection

*This manuscript describes a secondary analysis of 2 cross-sectional surveys that were conducted as part of a program evaluation of an integrated infant and young child feeding (IYCF) and micronutrient powder (MNP) intervention that included anemia as a primary outcome* [11]. The household surveys were conducted in representative samples of children aged 6–23 mo in Kapilvastu and Achham districts [11]. The first survey (from baseline) was conducted from December 2012 to February 2013, and the second survey was conducted from January 2016 to February 2016. Given that the prevalence of anemia in children aged 6–23 mo in both Kapilvastu and Achham remained relatively consistent between the 2 surveys (48.7%–47.5% in Kapilvastu and 32.6%–30.0% in Achham), we pooled the 2 surveys for the current analyses for each district and adjusted for the survey year (2013 compared with 2016) in all multivariable models. For both surveys, a two-stage cluster sampling method was used. Population proportion to size sampling selected 40 clusters from each district. A census in the selected clusters identified all children aged 6–23 *mo*. Using random sampling, 34 children from each cluster were selected in Kapilvastu and 33 in Achham without replacement for refusals or for clusters with less than the needed number of children.

The survey field teams participated in 2 wk of classroom and practical training, including standardization exercises for length and weight, and 3 d of pilot testing of all survey procedures in clusters in nearby districts not involved in the impact evaluation. Children’s length was measured to the nearest 0.1 cm using an Infant/Child/Adult ShorrBoard (Weigh and Measure LLC), and weight to the nearest 0.01 kg was measured using a Seca 874 Digital Floor Scale with Mother/Child function (SECA GmbH). Trained laboratory technicians collected venous blood specimens from children into blue top (zinc free) and purple top tubes, and then, they assessed the samples for hemoglobin concentration, malaria, and *H. Pylori* at the household using the HemoCue Hb-301 photometer, the malaria antigen (HRP2/pLDH) combination rapid diagnostic kit for *Plasmodium falciparum* and *P. vivax*, and the *H. Pylori* QuickVue rapid test kit (endline survey only), respectively. The HemoCue Hb-301 photometer is self-calibrating, but laboratory technicians also performed additional quality control procedures at the beginning of each day using 3 levels of liquid controls (low, normal, and high; Eurotrol). At the time of the baseline survey (December–February), there was only 1 positive malaria case, and at the time of the endline survey (January–February), there were only 3 positive malaria cases and no positive cases of *H. Pylori*. The laboratory technicians placed the blood specimens in a cold box containing frozen gel packs and a thermometer.

At a central portable laboratory established in the field in each cluster, the specimens were centrifuged within 1–2 h of collection, then transferred into cryovials, and stored in portable freezers. At the end of each day, the processed specimens were transferred to the freezers in the District Public Health Offices for storage until the end of data collection. At the end of the survey, all specimens were then transferred to the National Public Health Laboratory (NPHL) for storage at −86 °C. The specimens from NPHL were sent to the VitMin Lab, Willstaett, Germany, for the testing of ferritin, RBP, CRP, and α-1 acid glycoprotein (AGP) presented in this analysis using an in-house sandwich ELISA technique [12]. Jordan University of Science and Technology (Al Ramtha, Irbid, Jordan) analyzed the vitamin B12 and zinc specimens using an autoanalyzer and atomic absorption spectrophotometry, respectively.

Sample size calculations were designed to capture an approximate 10 percentage point (PP) public health significant difference for most biological indicators (total sample and among those without inflammation, where relevant) between the 2 surveys. For the endline survey, the final results of the baseline survey data were used to recalculate sample size estimates for the endline survey in order to capture an approximate 10 PP public health significant difference for most biological indicators (total sample and among those without inflammation, where relevant) between the 2 surveys. This resulted in changes in sample size estimates at endline, including a reduction in Achham.

### Ethical approval

Ethical approval was obtained from the Nepal Health Research Council for both the baseline and endline surveys. The CDC determined that this was a program evaluation and CDC staff participation was public health practice with no CDC investigators. The surveys complied with the Declaration of Helsinki revised in 1983. For each survey, interviewers described the purpose, procedures, risks, and benefits of the study and allowed the mothers to ask questions before inviting them and their children to participate in the survey. Mothers or other legal guardians then provided written informed consent to enroll themselves and their children in the study. If the mothers or guardians were illiterate, then a witness signature was obtained. Participants were informed that the data would be published. The results of hemoglobin and malaria testing were given to the participants at the time of data collection, and were explained to the participants in an understandable way. Those with severe anemia or malaria infection were referred to the health center. Survey participants also received a small towel, soap, nail cutter, and comb as a token of appreciation; they received these products even if they did not fully complete the survey. Data were stored securely, only classified staff had access, and the names and identifiers were separated from the main deidentified survey database after the completion of data collection.

### Data preparation

Each child’s hemoglobin was adjusted for altitude based on the GPS coordinates of the household using the 2011 WHO recommendations [2]. Anemia was defined as an altitude-adjusted hemoglobin concentration of <110 g/L. Moderate or severe anemia was defined as an altitude-adjusted hemoglobin concentration of <100 g/L; children with moderate and severe anemia were combined into a single category because only 6 and 7 children in the baseline and endline surveys, respectively, were classified as severely anemic (hemoglobin concentration < 70 g/L). Serum ferritin was adjusted for inflammation (CRP and AGP) using the Biomarkers Reflecting Inflammation and Nutritional Determinants of Anemia (BRINDA) linear regression technique with internal data-driven reference levels [13]. Iron deficiency was defined as an inflammation-adjusted serum ferritin concentration of <12 μg/L. Iron deficiency anemia was defined as iron deficiency plus anemia. Vitamin A deficiency was defined as an inflammation-adjusted serum RBP concentration of <0.58 μmol/L. A random sample of 485 children had their serum retinol measured. In a simple linear regression, a noninflammation-adjusted serum RBP concentration of 0.58 μmol/L corresponded with a noninflammation-adjusted serum retinol concentration of 0.70 μmol/L, the WHO defined cut-off for vitamin A deficiency [14]. Inflammation adjustment was performed based on the BRINDA linear regression technique [13]. Vitamin B12 deficiency was defined as a serum B12 concentration of < 203 pg/mL [15] without adjustment for inflammation [16]. Zinc deficiency was defined as a serum zinc concentration of <65 μg/dL collected in the morning and that of <57 μg/dL collected in the afternoon [17]. The correlation between plasma zinc and inflammation was assessed; given the lack of a negative association, no inflammation adjustment was applied to zinc concentration [18].

Length-for-age (LAZ) and weight-for-length (WLZ) were calculated using the WHO 2006 growth standards [18]. Stunting and wasting were defined as <−2 SDs for LAZ and WLZ, respectively. In accordance with WHO recommendations, all extreme LAZ (<−6 or >6) and WLZ (<−5 or >5) values were set to missing [15]. IYCF indicators were calculated based on the 2021 WHO/UNICEF indicators [19]. The minimum dietary diversity (MDD) is defined as the percentage of children aged 6–23 *mo* who consumed foods and beverages from ≥5 of 8 defined food groups during the previous day (breast milk; grains, roots, and tubers; pulses, nuts and seeds; dairy products; flesh foods; eggs; vitamin A–rich fruits and vegetables; and other fruits and vegetables). The minimum meal frequency (MMF) is defined as the percentage of children aged 6–23 *mo* who consumed solid, semisolid, or soft foods at least twice for breastfed infants aged 6–8 *mo*, ≥3 times for breastfed children aged 9–23 *mo*, and ≥4 times for nonbreastfed children (including milk feeds for nonbreastfed infants). The minimum acceptable diet (MAD) is defined as a combination of MDD and MMF.

### Statistical analyses

**FIGURE 1:**
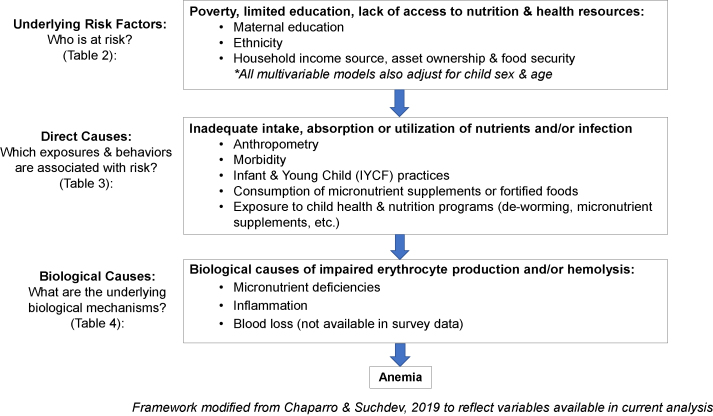
Conceptual framework of risk factors for anemia in children aged 6–23 mo in Kapilvastu and Achham districts, Nepal.

Frequencies and means with standard errors are presented from survey procedures accounting for clusters. Given that the prevalence of anemia was notably different in the 2 districts and that the risk factors for anemia in the 2 districts were hypothesized to vary substantially, we modeled the 2 districts separately. To estimate univariable and multivariable risk ratios, we used log-binomial regression models accounting for correlated errors within clusters using an exchangeable covariance structure [20,21]; when the log-binomial model did not converge, a log-Poisson model was used [22]. We first modeled the association of all predictors with anemia in univariable models. To determine which variables should be retained in multivariable models, we developed a causal framework for conceptualizing the anemia risk based on the variables in our dataset (Figure 1). This framework is based on that developed by Chaparro and Suchdev [8], which presents different levels for underlying risk factors, direct risk factors, and biological physiological causes for anemia. For our multivariable models, we modeled risk factors and potential causes of anemia for each risk “level” separately to prevent the adjustment of variables on the causal pathway linking exposures to anemia [23]. For example, in multivariable models for sociodemographic risk factors, such as maternal education, we did not want to adjust for infant feeding practices or iron deficiency, because we would expect these variables to be on the causal pathway linking maternal education to anemia, and the inclusion of these variables could attenuate the association between maternal education and anemia. For our sociodemographic characteristics, the goal was to identify which children are at an increased risk of anemia, as opposed to isolating an independent effect of each risk factor, and thus, it would have been inappropriate to adjust for variables on the causal pathway linking sociodemographic characteristics to anemia. For multivariable models for “underlying risk factors” for anemia (Table 3), models were adjusted for survey, child’s sex and age, and all underlying risk factors that are found to be significant in the multivariable models of underlying risk factors for anemia. In addition, we determined a priori that all multivariable models would adjust for the survey year (2013 compared with 2016) and child’s sex and age.

**TABLE 1:** Characteristics of survey participants in Kapilvastu and Achham districts, Nepal^1^

Characteristics	Kapilvastu	Achham	*P*
	*n* = 2488	*n* = 2221	
Survey year			<0.001
2013	1226 (49.3)	1226 (55.2)	
2016	1262 (50.7)	995 (44.8)	
Child’s age			0.22
6–11 mo	785 (31.6)	711 (32.0)	
12–17 *mo*	980 (39.4)	824 (37.1)	
18–23 *mo*	723 (29.1)	686 (30.9)	
Sex			0.94
Male	1317 (52.9)	1178 (53.0)	
Female	1171 (47.1)	1043 (47.0)	
Ethnicity			<0.0001
Upper ethnic caste	360 (14.5)	1493 (67.2)	
Dalit	410 (16.5)	703 (31.7)	
Other	1718 (69.1)	25 (1.1)	
Maternal education			<0.0001
No formal education	1296 (52.1)	1358 (61.2)	
Primary education	475 (19.1)	269 (12.1)	
Secondary or higher	716 (28.8)	593 (26.7)	
Main source of household income			<0.0001
Agriculture	1517 (61.0)	1581 (71.2)	
Remittance	253 (10.2)	344 (15.5)	
Other	718 (28.9)	296 (13.3)	
Household food security level			<0.0001
Food secure	1496 (60.1)	856 (38.5)	
Mildly food insecure	219 (8.8)	299 (13.5)	
Moderately food insecure	635 (25.5)	632 (28.5)	
Severely food insecure	138 (5.6)	434 (19.5)	
Household assets quartile^2^			0.12
Quartile 1 (more assets)	601 (24.2)	558 (25.1)	
Quartile 2	647 (26.0)	510 (23.0)	
Quartile 3	620 (24.9)	579 (26.1)	
Quartile 4 (fewer assets)	620 (24.9)	574 (25.8)	
Altitude (m)	94.6 ± 2.8	1284.2 ± 38.7	<0.001
<1000 m	2488 (100.0)	349 (15.7)	
1000–1500 m		1402 (63.1)	
1500–2000 m		469 (21.1)	
2000–2500 m		1 (0.05)	
Stunting (length-for-age < −2 SD)	973 (39.2)	1072 (48.5)	<0.001
Wasting (weight-for-length < −2 SD)	337 (13.6)	194 (8.8)	<0.001
Hemoglobin concentration (raw)	10.94 ± 0.05	11.64 ± 0.04	<0.001
Hemoglobin concentration (altitude-adjusted)^3^	10.94 ± 0.05	11.41 ± 0.03	<0.001
Anemia (all levels of severity)^3^	1197 (48.1)	698 (31.4)	<0.001
Mild anemia	788 (31.7)	550 (24.8)	
Moderate anemia	400 (16.1)	144 (6.5)	
Severe anemia	9 (0.4)	4 (0.2)	
Iron deficiency^4^	1344 (55.9)	1015 (47.7)	<0.001
Vitamin A deficiency^5^	183 (7.6)	96 (4.51)	<0.001
Vitamin B12 deficiency^6^	584 (47.3)	376 (39.9)	<0.001
Zinc deficiency^7^	454 (21.2)	516 (27.0)	0.003
Inflammation: CRP > 5 mg/L^8^	401 (16.7)	396 (18.6)	0.10
Inflammation: AGP > 1 mg/mL^8^	840 (34.9)	767 (36.0)	0.43

Remittance: income from a family member who has migrated. AGP, α-1 acid glycoprotein.

1 For categorical variables, values are in frequencies *n* (%)with *P* values from chi-squared tests. For continuous variables, values are expressed as mean ± standard error with *P* values from *t*-tests.

2 Household asset quartile developed using a principal component analysis for each district separately.

3 Altitude adjustment and anemia cut-offs were defined based on WHO recommendations [2]. Anemia is defined as an altitude-adjusted hemoglobin concentration of <11 g/dL; mild anemia is defined as an altitude-adjusted hemoglobin concentration of 10.0–10.9 g/dL, moderate anemia as that of 7.0–9.9 g/dL, and severe anemia as that of <7.0 g/dL.

4 Iron deficiency was defined as a serum ferritin concentration of <12 μg/L after inflammation adjustment using the BRINDA linear regression technique [13].

5 Vitamin A deficiency is defined as an inflammation-adjusted serum RBP concentration of <0.58 μmol/L. A random sample of 485 children had their serum retinol measured. In a simple linear regression, a noninflammation-adjusted serum RBP concentration of 0.58 μmol/L corresponded with a noninflammation-adjusted serum retinol concentration of 0.70 μmol/L; WHO defined the cut-off for vitamin A deficiency [14]. Inflammation adjustment was performed based on the BRINDA linear regression technique [13].

6 Vitamin-B12 deficiency is defined as a serum B12 concentration of <203 pg/mL,^17^ without adjustment for inflammation [15].

7 Zinc deficiency was defined as a serum zinc concentration of <65 μg/dL if assessed in the morning and of <57 μg/dL in the afternoon [16]. The correlation between plasma zinc concentration and inflammation was assessed; given the lack of a negative association, no inflammation adjustment was applied to zinc concentration [17].

8 Inflammation defined as an AGP concentration of >1 mg/gL or a CRP concentration of >5 mg/L [51,52].

**TABLE 2:** Underlying risk factors for anemia in children aged 6–23 *mo* in Kapilvastu and Achham districts, Nepal

Characteristics	Kapilvastu				Achham			
	Total *n* = 2488	Prevalence of anemia	Multivariable PR (95% CI)^1^^,^^2^	*P*	Total *n* = 2221	Prevalence of anemia	Multivariable PR (95% CI)^1^^,^^2^	*P*
Sociodemographic variables								
Survey year								
2013	1226	597 (48.7)	Reference		1226	400 (32.6)	Reference	
2016	1262	600 (47.5)	0.98 (0.88, 1.09)	0.70	995	298 (30.0)	0.95 (0.81, 1.11)	0.49
Child’s age								
6–11 *mo*	785	366 (46.6)	0.98 (0.87, 1.10)	0.69	711	245 (34.5)	1.42 (1.20, 1.68)	<0.001
12–17 *mo*	980	492 (50.2)	1.07 (0.96, 1.19)	0.22	824	287 (34.8)	1.44 (1.22, 1.70)	<0.001
18–23 *mo*	723	339 (46.9)	Reference		686	166 (24.2)	Reference	
Sex								
Male	1317	670 (50.9)	1.12 (1.03, 1.22)	0.01	1178	385 (32.7)	1.08 (0.97, 1.21)	0.16
Female	1171	527 (45.0)	Reference		1043	313 (30.0)	Reference	
Ethnicity								
Upper ethnic caste	360	123 (34.2)	Reference		1493	446 (29.9)	Reference	
Dalit	410	207 (50.5)	1.28 (1.09, 1.50)	0.002	703	245 (34.9)	1.14 (0.98, 1.33)	0.09
Other	1718	867 (50.5)	1.28 (1.13, 1.44)	<0.001	25	7 (28.0)	0.94 (0.50, 1.78)	0.85
Maternal education								
No formal education	1296	674 (52.0)	1.08 (0.96, 1.21)	0.20	1358	450 (33.1)	1.05 (0.89, 1.24)	0.55
Primary education	475	234 (49.3)	1.08 (0.96, 1.21)	0.19	269	86 (32.0)	1.03 (0.81, 1.31)	0.81
Secondary or higher	716	289 (40.4)	Reference		593	162 (27.3)	Reference	
Main household income source								
Agriculture	1517	745 (49.1)	Reference		1581	518 (32.8)	Reference	
Remittance	253	98 (38.4)	0.89 (0.77, 1.03)	0.10	344	101 (29.4)	0.89 (0.74, 1.06)	0.20
Other	718	354 (49.3)	1.02 (0.94, 1.11)	0.63	296	79 (26.7)	0.85 (0.69, 1.05)	0.13
Household food security level								
Food secure	1496	694 (46.4)	Reference		856	250 (29.2)	Reference	
Mildly food insecure	219	110 (50.2)	1.05 (0.91, 1.21)	0.54	299	102 (34.1)	1.08 (0.89, 1.31)	0.43
Moderately food insecure	635	317 (49.9)	0.95 (0.86, 1.05)	0.35	632	197 (31.2)	0.97 (0.95, 1.10)	0.59
Severely food insecure	138	76 (55.1)	1.04 (0.88, 1.22)	0.66	434	149 (34.3)	1.07 (0.89, 1.27)	0.49
Household assets quartile^3^								
Quartile 1 (more assets)	601	282 (46.9)	Reference		558	127 (22.8)	Reference	
Quartile 2	647	295 (45.6)	0.96 (0.84, 1.11)	0.60	510	173 (33.9)	1.48 (1.19, 1.83)	<0.001
Quartile 3	620	275 (44.4)	0.93 (0.82, 1.06)	0.28	579	207 (35.8)	1.49 (1.21, 1.84)	<0.001
Quartile 4 (fewer assets)	620	345 (55.7)	1.13 (0.98, 1.30)	0.10	574	191 (33.3)	1.33 (1.03, 1.72)	0.03

Remittance: income from family member who has migrated. PR, prevalence ratio.

1 Prevalence given as *n* (%); PR and 95% CI from generalized linear models with a log link and binomial distribution, accounting for correlated errors within cluster with an exchangeable covariance structure.

2 All multivariable models adjusted for the survey year (2013 compared with 2016), child’s sex and age, and any additional sociodemographic characteristics found to be significant at the *P* < 0.1 level in the univariable models.

3 Household asset quartile developed using a principal component analysis for each district separately

For multivariable models for “direct causes” of anemia, models were adjusted for survey, child’s sex and age, and all underlying risk factors that are found to be significant in the multivariable models in Table 2. All “direct cause” variables found to be significant at the *P* < 0.1 level in the univariable model with sufficient distribution in the response (at least *n* = 25 participants in each response category) were included in the multivariable model. In sensitivity analyses, we also assessed additional co-linear anthropometric, morbidity, and IYCF variables in separate multivariable models, including separate models replacing anthropometric *z*-scores with binary outcomes, models that replaced “any morbidity” with individual morbidities (fever, diarrhea, and cough), and models that replaced the MMF with the MAD.

**TABLE 3:** Direct risk factors for anemia in children aged 6–23 *mo* in Kapilvastu and Achham districts, Nepal

Characteristics	Kapilvastu				Achham			
	*n*	Prevalence of anemia	Multivariable PR (95% CI)^1^^,^^2^	*P*	*n*	Prevalence of anemia	Multivariable PR (95% CI)^1^^,^^2^	P
Anthropometry								
LAZ (anemic vs. nonanemic: mean ± SE)	1194 vs. 1287	−1.77 ± 0.06 vs. −1.61 ± 0.06	0.98 (0.95, 1.01)	0.24	698 vs. 1523	−1.85 ± 0.04 vs. −2.00 ± 0.06	0.93 (0.89, 0.97)	0.001
WLZ (anemic vs. nonanemic: mean ± SE)	1195 vs. 1285	−0.94 ± 0.05 vs. −0.78 ± 0.05	0.96 (0.92, 1.00)	0.03	696 vs. 1513	−0.74 ± 0.04 vs. −0.74 ± 0.03		
Morbidity								
Diarrhea, fever, or cough in the previous 2 wk	1498	763 (50.9)	1.12 (1.04, 1.21)	0.001	1452	470 (32.4)		
No morbidity	990	434 (43.8)	Reference		769	228 (29.7)		
Infant and young child feeding (IYCF): consumption in the last 24 h								
Minimum dietary diversity^3^	754	323 (42.8)	0.93 (0.83, 1.04)	0.17	655	184 (28.1)	0.96 (0.86, 1.07)	0.47
No minimum dietary diversity	1734	874 (50.4)	Reference		1566	514 (32.8)	Reference	
Minimum meal frequency^3^	1216	568 (46.7)			1260	367 (29.1)	0.94 (0.82, 1.07)	0.32
No minimum meal frequency	1149	568 (49.4)			901	310 (34.3)	Reference	
Store-bought fortified food	170	55 (32.4)	0.75 (0.60, 0.94)	0.01	118	30 (25.4)	0.88 (0.62, 1.22)	0.42
Did not consume fortified food	2127	1051 (49.4)	Reference		1999	634 (31.7)	Reference	
Health and nutrition programs								
Iron syrup in last 7 d	21	9 (42.9)			41	7 (17.1)		
No iron syrup in the previous 7 d	2467	1188 (48.2)			2180	691 (31.7)		
Vitamin A supplementation in the past 6 *mo*	2003	967 (48.3)			1936	604 (31.4)		
No vitamin A in the past 6 *mo*	485	230 (47.4)			285	94 (33.0)		
Deworming in the past 6 mo	1356	671 (49.5)	1.09 (0.98, 1.20)	0.11	1524	461 (30.3)		
No deworming in the past 6 *mo*	1132	526 (46.5)	Reference		697	237 (34.0)		
No receipt of MNP for child	1792	874 (48.8)	Reference		1476	485 (32.9)	Reference	
Mother received 1 distribution	434	222 (51.2)	1.02 (0.90, 1.15)	0.79	374	126 (33.7)	0.97 (0.74, 1.27)	0.81
Mother received ≥2 distributions	262	101 (38.6)	0.79 (0.63, 0.98)	0.03	371	87 (23.5)	0.71 (0.50, 1.00)	0.0496
Received nutritious flour for children or pregnant women in the last 12 *mo*	55	28 (50.9)			93	24 (25.8)	0.74 (0.54, 1.01)	0.06
No nutritious flour	2433	1169 (48.1)			2128	674 (61.67)	Reference	

LAZ, length-for-age *z*-score; Min, minimum; MNP, micronutrient powder; PR, prevalence ratio; WLZ, weight-for-length *z*-score.

1 Prevalence given as *n* (%); PR and 95% CI from generalized linear models with a log link and binomial distribution, accounting for correlated errors within cluster with an exchangeable covariance structure.

2 All multivariable models adjusted for the survey year (2013 compared with 2016), child’s sex and age, and demographic characteristics significant at the *P* < 0.05 level in multivariable models in Table 2. Multivariable models also contain any “direct cause” variable found to be significant at the *P* < 0.10 level in the univariable model with the exception of co-linear variables specified below.

3 Minimum acceptable diet was input into a separate multivariable model from minimum meal frequency and minimum diet diversity. The minimum dietary diversity is defined as the percentage of children aged 6–23 *mo* who consumed foods and beverages from ≥5 of 8 defined food groups during the previous day (breast milk; grains, roots, and tubers; pulses, nuts and seeds; dairy products; flesh foods; eggs; vitamin A–rich fruits and vegetables; and other fruits and vegetables). The minimum meal frequency is defined as the percentage of children aged 6–23 *mo* who consumed solid, semisolid, or soft foods at least twice for breastfed infants aged 6–8 *mo*; ≥3 times for breastfed children aged 9–23 *mo*; and ≥4 times for nonbreastfed children (including milk feeds for nonbreastfed infants).

Univariable log-binomial models for anemia were also built with biomarkers of micronutrient deficiencies and inflammation as predictors. The multivariable models for “biologic causes” were adjusted for survey, child’s sex and age, underlying risk factors (ethnicity in Kapilvastu and household asset tertile in Achham), and other individual biomarkers (from Table 4) that were found to be significant in the univariable models. We calculated the average attributable fraction (AF) for the population with anemia for all biomarkers that were found to be significant (*P* < 0.05) in the multivariable models. Average AFs were calculated using the method proposed by Eide and Gefeller [24] using a modified version of the SAS macro developed by Ruckinger [25]. We modified the analysis to account for survey clusters and adjusted multivariable models for covariates: child’s sex and age and underlying risk factors (ethnicity and in Kapilvastu and household asset tertile in Achham). The average AF was calculated with the stepwise removal of exposures from a multivariable model, and is the average of all sequential AFs for this exposure over all possible removal orderings. We selected this method over Levin’s AF methodology, given that the average AF allows one to sum the AF for multiple exposures without exceeding 100% [24,26]. We report the AF results as anemia associated with micronutrient deficiencies or inflammation and do not describe them as “attributable” because we cannot make true causal inference with cross-sectional data.

**TABLE 4:** Prevalence estimates and attributable fractions for the biological causes of anemia in children aged 6–23 mo in Achham and Kapilvastu districts, Nepal

Characteristics	Kapilvastu					Accham				
	Total *n* = 2406	Prevalence of anemia^1^	Multivariable PR (95% CI)^2^	*P*	Average attributable fraction^3^	Total *n* = 2129	Prevalence of anemia^1^	Multivariable PR (95% CI)^2^	*P*	Average attributable fraction^3^
Micronutrient deficiencies										
Iron deficiency^4^	1344	802 (59.7)	1.76 (1.57, 1.99)	<0.001	32.1%	1015	450 (44.3)	2.24 (2.00, 2.51)	<0.001	28.2%
No iron deficiency	1062	359 (33.8)	Reference			1114	219 (19.7)			
Vitamin A deficiency^5^	183	107 (58.5)	1.12 (0.97, 1.30)	0.13		96	43 (44.8)	1.22 (0.94, 1.58)	0.14	
No vitamin A deficiency	2223	1054 (47.4)	Reference			2033	626 (30.8)			
Vitamin B12 deficiency^6^	913	457 (50.1)				701	229 (32.7)			
No B12 deficiency	1414	688 (48.7)				1306	411 (31.5)			
Zinc deficiency^7^	454	253 (55.7)	1.13 (1.02, 1.26)	0.02	4.2%	516	184 (35.7)			
No zinc deficiency	1690	815 (48.2)	Reference			1396	438 (31.4)			
Inflammation^8^										
CRP > 5 mg/L	401	231 (57.6)	1.21 (1.09, 1.34)	<0.001	2.4%	396	195 (49.2)	1.54 (1.31, 1.81)	<0.001	12.0%
CRP ≤ 5 mg/L	1996	924 (46.3)	Reference			1732	473 (27.3)	Reference		
AGP > 1 mg/mL	840	460 (54.8)	1.16 (1.05, 1.27)	0.003	2.5%	767	319 (41.6)	1.32 (1.15, 1.52)	<0.001	7.8%
AGP ≤ 1 mg/mL	1566	701 (44.8)	Reference			1362	350 (25.7)	Reference		

AGP, α-1 acid glycoprotein; PR, prevalence ratio.

1 Prevalence expressed as *n* (%); PR and 95% CI from generalized linear models with a log link and binomial distribution, accounting for correlated errors within cluster with an exchangeable covariance structure. When the model did not converge, a Poisson distribution was used.

2 Biomarkers found to be significant (*P* < 0.05) in the univariable models are included in the multivariable models. All multivariable models also adjust for survey (baseline compared with endline), child’s sex and age, and demographic characteristics significant at the *P*<0.05 level in multivariable models in table 2 (ethnicity in Kapilvastu and asset quartile in Achham).

3 Attributable fractions were calculated for all biomarkers found to be significant (*P* < 0.05) in the multivariable models. Average AFs were calculated using the method proposed by Eide and Gefeller [24]. The average AF is the average of all AFs estimated from stepwise removal of exposures from a multivariable model over all possible removal orderings.

4 ron deficiency is defined as a serum ferritin concentration of <12 μg/L after inflammation adjustment using the BRINDA linear regression technique [13,53].

5 Vitamin A is deficiency defined as an inflammation-adjusted serum RBP concentration of <0.58 μmol/L. A random sample of 485 children had their serum retinol measured. In a simple linear regression, a noninflammation-adjusted serum RBP concentration of 0.58 μmol/L corresponded with a noninflammation-adjusted serum retinol concentration of 0.70 μmol/L, which is the WHO defined cut-off for vitamin A deficiency [14]. Inflammation adjustment performed based on the BRINDA linear regression technique [13].

6 Vitamin B12 deficiency is defined as a serum B12 concentration of <203 pg/mL [18], without adjustment for inflammation [15].

7 Zinc deficiency is defined as a serum zinc concentration of <65 μg/dL if assessed in the morning and that of <57 μg/dL in the afternoon [16]. Correlation between plasma zinc and inflammation was assessed; given the lack of a negative association, no inflammation adjustment was applied to zinc concentration [17].

8 Inflammation is defined as a AGP concentration of > 1 mg/gL or a CRP concentration > 5 mg/L [51,52].

## Results

Sociodemographic characteristics of the participants from the 2 districts are notably different (Table 1). The prevalence of food insecurity in Kapilvastu was 39.9% compared with 61.5% in Achham, with 19.5% of the households in Achham reporting severe food insecurity relative to only 5.6% in Kapilvastu. The mean altitude of households in Kapilvastu was 94.6 m above sea level and 1284 m in Achham; 84.0% of the participants in Achham lived at an altitude of >1000 m, and thus, altitude adjustments were needed in their hemoglobin for anemia assessment, as per WHO recommendations [2]. The prevalence of both anemia and micronutrient deficiencies (except zinc) was higher in the Kapilvastu district than in Achham. In both districts, the prevalences of inflammation, as assessed with elevated CRP and AGP, were approximately one-sixth and one-third, respectively.

The associations between different sociodemographic characteristics and anemia were notably different in the 2 districts (Table 2). In multivariable models, male children and children who were not from an upper ethnic caste had an increased risk of having anemia in Kapilvastu. In Achham, younger children and children from households with fewer assets had an increased risk of anemia. Although older children (18–23 mo) in Accham had a decreased risk of anemia (relative to 6–11 mo or 12–17 mo), anemia was persistently between 46.6% and 50.2% in all age groups from 6 to 23 *mo* in Kapilvastu.

In multivariable analyses of the direct causes of anemia in Kapilvastu (Table 3), we found that morbidity (diarrhea, fever, or cough) in the previous 2 wk was associated with an increased risk of having anemia, whereas higher weight-for-length *z*-score (WLZ), consumption of store-bought fortified foods in the previous day, and receipt of ≥2 MNP distributions were all associated with a reduced risk of anemia. In the sensitivity analysis replacing WLZ with wasting, we also found that wasting was associated with an increased risk of anemia [adjusted PR (95%CI): 1.13 (1.02, 1.25); *P* = 0.02]. In Achham, only the higher length-for-age *z*-score and receipt of ≥2 MNP distributions were significantly associated with a reduced risk of anemia, and receipt of nutritious flour for children or pregnant women in the last 12 *mo* was marginally associated with reduced risk (*P* = 0.06). In sensitivity analyses, we did not find a significant association between any of the individual food groups or individual causes of morbidity (diarrhea, fever, or cough) and anemia in either district.

In multivariable models of the biological causes of anemia, we found that children with iron deficiency had 1.76 [95% CI: (1.57, 1.99)] times risk of having anemia in Kapilvastu and 2.24 [95% CI: (1.97, 2.51)] times risk of having anemia in Achham (Table 4). In AF analyses, we estimated that the proportion of anemia associated with iron deficiency was 28.2% in Achham and 32.1% in Kapulvastu. Zinc deficiency was also significantly associated with anemia in Kapilvastu, but not in Achham. Inflammation was significantly associated with an increased risk of having anemia in both districts, with an estimated additional risk of anemia associated with inflammation (CRP or AGP) of one-fifth in Achham and 5% in Kapilvastu.

## Discussion

In this analysis of large, representative samples in 2 different districts in Nepal, we identified several underlying and direct risk factors, as well as biologic causes of anemia, and also estimated the proportion of anemia associated with several biological causes. Among the potential biological causes, iron deficiency was highly prevalent (55.9% in Kapilvastu and 47.7% in Achham) and was strongly associated with anemia in both districts. We also found that inflammation was significantly associated with an increased risk of anemia in both districts, and zinc deficiency was associated with an increased risk of anemia in Kapilvastu.

Despite differences by district, WHO considers the prevalence of iron deficiency of ≥40% to be a “high” public health problem; thus, iron deficiency in both Kapilvastu and Achham is a severe public health problem [27]. Iron deficiency in early life can result in the prioritization of erythropoiesis over the brain [28], despite the critical role that iron plays in brain development. Iron deficiency in early life has consistently been shown to have life-long consequences for neurocognitive development [[29], [30], [31], [32], [33], [34]] and may also influence immune development and vaccination response in early childhood [35]. Addressing iron deficiency in young children in Nepal, and other contexts with a high prevalence of deficiency, is critical to prevent anemia and protect the developmental potential of young children.

We observed substantial differences in the prevalences of anemia and iron deficiency, and those of important underlying and direct risk factors for anemia in the 2 districts studied. In the Accham district, where the prevalence of anemia was 31.4%, significant predictors included child’s age, household asset ownership, and length-for-age *z*-score. In Kapilvastu, where the prevalence of anemia was 48.1%, significant predictors included child’s sex and ethnicity, wasting, and weight-for-length *z*-scores, any morbidity in the previous 2 wk, consumption of store-bought fortified foods, and receipt of at least 2 MNP distributions. We also found notable heterogeneity in the contribution of inflammation to anemia in young children in the 2 districts (4.9% in Kapilvastu compared with 19.8% in Achham). The data were collected during the winter months, so it is possible that the higher altitude and colder temperatures in Accham contributed to increased time indoors and smoke exposure, differential exposure to livestock, and infectious diseases. Interestingly, the prevalence of food insecurity in Accham was higher, but the prevalence of anemia and several micronutrient deficiencies were lower. Notably, the asset quartile was a significant predictor of anemia in Achham, whereas food security was not, indicating that the self-report of the household food insecurity scale may not capture food security well in this sample. Furthermore, in a previous work in Achham and Kapilvastu, we found that mothers in Achham tended to have more frequent contact with their female community-health volunteers (FCHVs) and a higher uptake of nutrition interventions [36], so this may also explain the higher prevalence of micronutrient deficiencies despite lower food insecurity. Taken together, our findings highlight the importance of multipronged approaches to anemia both inside and outside of the nutrition sector—including strengthening infection control, water, sanitation and handwashing, education, and household economic resources that are important drivers of anemia reduction, and continuing to include effective programs to control iron and other micronutrient deficiencies and improve the diet quality of young children [[37], [38], [39]].

Coverage of iron and micronutrient interventions was relatively low in these 2 districts; however, some program indicators were associated with a lower risk of having anemia. For example, in Kapilvastu, children who consumed store-bought fortified foods in the previous day (6.8% of children) had a lower risk of anemia than children who had not consumed fortified foods. Similarly, children of caregivers who received ≥2 MNP distributions had a significantly lower risk of having anemia in both districts, and receipt of nutritious flour for children or pregnant women in the last 12 *mo* was also associated with a marginally significant decreased risk of having anemia in Achham (*P* = 0.06). We previously reported that in the endline evaluation, 20% of the caregivers in Kapilvastu and 35% in Achham had received MNP multiple times during the program and that frequent interactions with FCHVs were associated with increased MNP coverage and IYCF optimal practices [36,40]. Continuing to strengthen iron and micronutrient interventions, particularly at the community level, has the potential to further reduce anemia risk [41,42].

The prevalence of anemia in Achham in our survey is similar to the 2016 Nepal National Micronutrient Status Survey (NNMSS), which estimated a 33% national prevalence of anemia in children 6–23 mo [10]; our estimated prevalence in Kapilvastu is higher than that in the NNMSS. The NNMSS, which was not powered for district or regional estimates stratified by age, estimated that among children aged 6–59 mo, 15% of children in the hill’s ecological zone (where Achham is located) and 23% in the Terai (where Kapilvastu is located) had anemia, which corresponds to the district-level patterns that we also observed for higher prevalence in Kapilvastu. Our analyses and the NNMSS analyses used the 2011 WHO adjustment for hemoglobin, although WHO is currently re-evaluating hemoglobin cut-offs and altitude adjustments. Altitude-adjustment was unlikely to have had a large effect on the anemia prevalence in Kapilvastu because all participants lived at an altitude of <500 m above sea level; however, in Achham, where the altitude ranged from 597 to 2085 m, it is possible that there was underadjustment for participants residing at lower altitudes [43]. The 2016 Demographic and Health Survey (DHS) reported a much higher prevalence of 68.7% in children aged 6–23 mo and 52.7% in children aged 6–59 mo [44]. However, DHS uses a single drop of blood analyzed on the Hemocue 201, whereas the NNMSS used venous blood and measured hemoglobin on the Hemocue 301. It has been shown that hemoglobin assessed using capillary drops varies from that assessed using venous blood [[45], [46], [47]] and that the venous blood sample is the preferred blood source over capillary samples. Furthermore, data were collected in different seasons for the NNMSS and DHS.

Despite differences in prevalence estimates, our analysis of risk factors identified several similar risk factors in analyses from the national data [10,48,49]. In the NNMSS, age, ethnicity, recent fever, MNP intake, inflammation (ln CRP), RBP, and glucose-6-phospate dehydrogenase were all significant predictors of anemia in children aged 6–59 *mo* [10]. In the DHS, underweight children, and/or children whose mothers have no formal education, children who were not in the highest socioeconomic class (based on asset ownership), and children living in the Terai ecological region had an increased risk of anemia [49]. Our analyses, which are from large district-level surveys representative of children aged 6–23 *mo*, also found differential associations between risk factors and anemia in the 2 districts.

We estimate that in children aged 6–23 *mo*, the proportions of anemia that are associated with iron deficiency are 28.2% and 32.1% in Achham and Kapilvastu districts, respectively. Our estimate is in line with research from Ko et al. [26] who estimated that the proportion of anemia in children aged 6–59 *mo* that was associated with iron deficiency in Nepal based on the NNMSS ranged from 32% to 52% depending on the method used to calculate the AF. The higher estimates in Ko et al’s [26] analysis were derived from Levin’s approach using logistic regression ORs, which are only appropriate when the outcome is rare, and have been shown to produce higher estimates than other AF methods [25]. Interestingly, Ko et al. [26] found that the contribution of iron deficiency to anemia among preschool children was higher in Nepal than in other countries, where the AF ranged from 2% to 52% depending on underlying malaria and inflammation prevalence, as well as the methodology used to calculate the AF.

Nepal is unique in the opportunity to examine predictors of anemia using diverse datasets, including variables of the underlying risk factors, intermediate risk factors, direct causes, and physiological mechanisms for anemia at both the national and district-specific levels, highlighting differential predictors of anemia across settings and age groups. Although this has been suggested as important for examining predictors to plan effective prevention and control strategies [50], it might be challenging for many countries and internally within countries due to cost and complexity. Furthermore, it is critical for existing programs to have effective quality-of-service access and anemia intervention delivery through the established platforms and to strengthen them if they are weak. The descriptive prevalence results (Table 1) identified multiple public health problems that justify public health action regardless of their associations with anemia, including reducing deficiencies of iron, vitamin B12, and zinc; reducing inflammation; improving nutritional status; and improving household food security.

Our study has its strengths and limitations. Strengths include the large, representative samples of children aged 6–23 *mo* from 2 districts in Nepal, allowing for more granular, district-level analyses of risk factors for having anemia, which usually is not possible in national surveys. Other strengths include the use of venous blood, which is the preferred blood source over capillary blood drops [[45], [46], [47]]; the assessment of multiple micronutrient and inflammation biomarkers, and the use of log-binomial regression models for prevalence ratios (which better reflect risk of having anemia than ORs from logistic regression when the outcome is prevalent). Our analyses are limited by the indicators assessed during the surveys, and thus, we were unable to include the important risk factors for anemia, such as genetic hemoglobin disorders or other infections, including hookworm and other soil-transmitted helminths that could cause blood loss. Of note, our surveys were conducted outside of the typical malaria season in Nepal, and thus, the 4 cases that we identified at baseline and endline did not contribute meaningfully to anemia risk. Seasonality could play an important role in anemia risk, particularly in Kapilvastu, which due to its lower elevation than Achham experiences more seasonal malaria. In addition, although risk factors for severe anemia may be different from those for mild anemia [9], due to the low prevalence of moderate and severe anemia in our surveys, we were unable to model anemia severity. The AF analyses were completed using cross-sectional data, and thus, we can neither make true causal inference on “risk” reduction of anemia nor eliminate the possibility of reverse causality. Furthermore, because our analysis used prevalence data, we could not distinguish between the factors associated with the development of anemia and the factors associated with the duration of anemia; hence, we framed the results as “risk of having anemia” and “risk of anemia,” respectively.

Our analysis of these 2 large cross-sectional surveys indicated that anemia, iron deficiency, vitamin B12 deficiency, and zinc deficiency are important public health problems in both Kapilvastu and Achham districts and that anemia and iron deficiency are strongly correlated. We also found that zinc deficiency (in Kapilvastu) and inflammation (in both districts) contribute to risk of having anemia, highlighting the need for continued attention to programs that increase iron intake among young children and for multisectoral approaches to prevent anemia and vitamin and mineral deficiencies and to improve nutritional status and household food security in young children in Nepal.

## Data Availability

The data described in the manuscript, code book, and analytic code will be made available upon request pending approval.
